# Effectiveness of immediate appointment scheduling in smoking cessation clinics for patients with chronic airway diseases: Preliminary results from a randomized trial

**DOI:** 10.18332/tid/191782

**Published:** 2024-08-23

**Authors:** Dilek Karadoğan, Tahsin Gökhan Telatar, İlknur Kaya, Siahmet Atlı, Neslihan Köse Kabil, Feride Marım, Merve Yumrukuz Şenel, Aycan Yüksel, Burcu Yalçın, Ökkeş Gültekin, Merve Erçelik, Metin Akgün

**Affiliations:** 1 Department of Chest Diseases, School of Medicine, Recep Tayyip Erdoğan University, Rize, Türkiye; 2 Department of Public Health, School of Medicine, Recep Tayyip Erdoğan University, Rize, Türkiye; 3 Department of Chest Diseases, Faculty of Medicine, Kutahya Health Sciences University, Kütahya, Türkiye; 4 Department of Chest Diseases, Van Education and Research Hospital, Health Sciences University, Van, Türkiye; 5 Department of Chest Diseases, Yalova Training and Research Hospital, Yalova, Türkiye; 6 Department of Chest Diseases, Faculty of Medicine, Balikesir University, Balıkesir, Türkiye; 7 Department of Chest Diseases, Faculty of Medicine, Başkent University, Ankara, Türkiye; 8 Department of Chest Diseases, Merzifon Karamustafapasa State Hospital, Amasya, Türkiye; 9 Department of Chest Diseases, Kemalpaşa State Hospital, İzmir, Türkiye; 10 Department of Chest Diseases, Faculty of Medicine, Süleyman Demirel University, Isparta, Türkiye; 11 Department of Chest Diseases, School of Medicine, Ağrı İbrahim Çeçen University, Ağrı, Türkiye

**Keywords:** smoking, asthma, COPD, smoking cessation, access

## Abstract

**INTRODUCTION:**

Patients with airway diseases who bear the burden of smoking need access to smoking cessation support. We aimed to investigate the impact of immediately scheduled appointments on access to smoking cessation clinics compared with usual care in this patient group.

**METHODS:**

This multicenter, prospective, randomized, open-label study was conducted between November 2022 and June 2023 at pulmonary outpatient clinics. The study included adult patients who were current smokers and had a diagnosis of asthma, COPD, or bronchiectasis for at least six months. Sequentially randomization was used for the allocation of patients in a 1:1 ratio to two study arms: the usual support arm (representing the current standard care procedure) and the immediate support arm (involving intensive brief cessation advice followed by the immediate arrangement of an appointment at the same clinic's smoking cessation service). After one week, both patient groups were contacted by phone to assess their quit attempts and whether they had sought assistance from smoking cessation outpatient clinics (SCCs).

**RESULTS:**

A total of 397 patients were enrolled in the study, with 199 allocated to the usual support arm and 198 allocated to the immediate support arm. Within the first week, 18.1% of patients in the usual support arm and 77.3% of patients in the immediate support arm sought assistance from the smoking cessation clinic (p<0.001). The rate of smokers without an intention to quit was 56.7% in the usual support arm and 27.7% in the immediate support arm in the first week of follow-up. Immediate appointment scheduling was significantly associated with a 13-fold (OR=13.38; 95% CI: 8.00–22.38) increase in referral rates in the multivariate logistic regression model.

**CONCLUSIONS:**

Arranging instant appointments has increased access to SCCs by 13 times compared to the usual care, this group of patients should be given an immediate appointment to SCCs.

## INTRODUCTION

Prevention of tobacco exposure in patients with asthma, COPD, and bronchiectasis, which are the most common chronic airway diseases, not only affects their etiopathogenesis but also plays a crucial role in disease progression and course. Smokers with COPD, in particular, face greater challenges in quitting smoking compared to smokers without COPD, with 40% of COPD patients continuing to smoke even after diagnosis^1^. Several factors contribute to this ongoing smoking status, including early smoking initiation, higher cigarette smoke inhalation volume, increased addiction scores, lower self-confidence and self-efficacy, and higher prevalence of accompanying conditions such as depression^1,2^. Similarly, the prevalence of current smoking among asthma patients is between 20–25%, similar to the general population^1^. Smokers with asthma exhibit higher nicotine dependence scores and participate less in educational programs compared to non-asthmatic smokers. Furthermore, recent studies have established a link between smoking exposure and the development of bronchiectasis^3^.

Although preventing tobacco exposure is vital in managing chronic airway diseases, real-life approaches for smoking cessation have shown limited success^4^. The current approach, following the Prochaska–DiClemente phase model, does not recommend initiation pharmacotherapy for smoking cessation until the patient reaches the preparation stage^5^. However, randomized controlled trials, such as the Lung Health Study, have demonstrated higher rates of smoking cessation when immediate cessation support, including the initiation of pharmacotherapy, is provided^4,6,7^. In certain countries, smoking cessation outpatient clinics typically operate through scheduled appointments and are primarily overseen by specialists in respiratory health^8,9^. However, due to the demanding nature of routine outpatient services, physicians often struggle to allocate sufficient time to initiate immediate cessation treatment for smoker outpatients’ nature of routine outpatient services, and so physicians often struggle to allocate sufficient time to initiate immediate cessation treatment for smoking outpatients. Therefore, the immediate initiation of smoking cessation pharmacotherapy and counseling may not always be feasible^7^. Instead, patients are advised to schedule appointments with existing smoking cessation clinics^10^. Unfortunately, individuals with chronic airway diseases, encountering these teachable moments, often lose their motivation due to such barriers and continue smoking.

In our previous study, we determined the smoking status of patients with chronic airway diseases in Turkiye^10^. We found that 34% of COPD patients and 18% of asthmatics were current smokers and they received routine smoking cessation brief interventions. After one month, 85.1% of current asthmatic smokers had not tried to call a quitline, while 14.8% had tried to contact a quitline, but none had contacted a smoking cessation clinic. Only 1.9% of COPD smokers visited a smoking cessation clinic^10^. Therefore, we planned this study on how to increase the access of these patients to smoking cessation services. The aim of this study is to investigate the effect of immediately scheduling smoking cessation appointments on access to smoking cessation services for patients with chronic airway diseases and to compare the outcomes with current practice.

## METHODS

### Settings and participants

This prospective, multicenter, randomized, open-label study, received ethical approval from the Institutional Review Board (Ethical Approval Number: 2022/09), and the study protocol was registered with clinical trials online as number (ClinicalTrials.gov Identifier: NCT05764343). The study was conducted in 10 different institutions with researchers serving in secondary and tertiary care chest diseases outpatient clinics, certified in smoking cessation programs of the Ministry of Health and members of the Turkish Thoracic Society Early Career Members Task Force Group. Data were collected from pulmonary outpatient clinics between November 2022 and June 2023, with the written informed consent of participants.

### Sample size calculation

The sample size was calculated using G*Power 3.1.9.7, with the aim of achieving a study power of 95% (1-β), an α of 0.05, and an anticipated effect size of 20%. This was based on the lack of comparable studies in this specific population. It was anticipated that the effect size would be substantial and, therefore, set between medium (d=0.5) and large (d=0.8) according to common interpretations of Cohen’s d^11^. Consequently, the minimum sample size was determined to be 325 participants, which should ensure adequate power to detect a significant difference given the expected effect, aligning with or exceeding typical thresholds used in similar studies.

### Inclusion and exclusion criteria

Inclusion criteria were defined as participants who were aged ≥18 years, had a diagnosis of asthma and/or COPD and/or bronchiectasis for at least six months according to electronic health records in light of recent guidelines^12,13^, were current smokers who smoked at least 100 cigarettes in their lifetime and continuing to smoke daily or on some days, were willing to participate in the study, and could be reached by telephone one week after randomization. On the other hand, participants with active psychiatric disorders and participants with impaired cognitive functioning were excluded.

### Interventions and randomization

Participants were selected from patients attending pulmonology outpatient clinics. Participants were sequentially allocated in a 1:1 ratio to the two arms, based on the order of presentation. The routine support arm reflects the current standard care procedure. The immediate support arm, on the other hand, involves intensive brief cessation advice followed by an immediate appointment with the smoking cessation service of the same clinic. Demographic and clinical information was also recorded. One group received a brief smoking cessation intervention and was recommended to seek assistance from smoking cessation outpatient clinics by scheduling appointments through quitlines, as per the routine practice. Patients allocated to the other group were immediately scheduled appointments at the smoking cessation outpatient clinic in addition to receiving the brief smoking cessation intervention. Both groups of patients were contacted by phone one week later to inquire about their quit attempts and whether they had sought help from smoking cessation outpatient clinics.

Nicotine dependence of the patients was assessed with the Fagerström test for nicotine dependence (FTND) and included in the analyses as a continuous variable^14^. The presence of anxiety and depression diagnoses were obtained from self-reports and confirmed from electronic patient records. Income status was obtained by asking the patient, and grouped according to the minimum wage. Presence of airway disease diagnoses at least 6 months were obtained from electronic patient records and verified according to guidelines^12,13^.

### Statistical analysis

Data were analyzed using IBM SPSS Statistics for Windows. Armonk, NY, USA, IBM Corp. software. The numerical data obtained in the study are shown with mean and standard deviation values. Categorical data are presented as frequencies and percentages. Relationships between categorical data were evaluated with the chi-squared test. The distribution characteristics of continuous data were determined by Kolmogorov Smirnov and Shapiro Wilk tests, and differences between groups were evaluated with the Mann Whitney U test. A multivariable logistic regression model was designed to evaluate the factors that have an effect on smoking cessation outpatient clinic admission. The multivariable logistic regression model included variables with p<0.05 in univariate analyses, and adjusted variables included not only biological probability but also variables that were not homogeneously distributed at randomization. Accordingly, the model was adjusted for income level, FEV1% value as a continuous number, FTND score, airway disease type such as asthma, COPD, or bronchiectasis, and education level. Results are presented as adjusted odds ratios (AORs) with 95% CI. In all statistical analyses, the level of significance was accepted as p<0.05, and all tests were two-tailed.

## RESULTS

Most of the patients had COPD and asthma, accounting for 55.4% and 41.5% of the cohort, respectively. The prevalence of bronchiectasis among patients was 3.02%. The mean age of the patients was 53 ± 13 years, with males comprising 67.2% of the population, 80.8% were married, 44.5% had completed primary school education, and 62% had an income level ≤ minimum wage. Additionally, 17.1% of the patients had a diagnosis of depression, 18.9% had an anxiety disorder diagnosis, and 48.1% had comorbidities. Among the COPD patients, 40.9% had Category B disease, while among those with asthma, the majority had mild to moderate severity levels (Table 1).

**Table 1:** Characteristics of study population and comparisons according to randomization arm. Multicenter, prospective, randomized, open-label study, conducted between November 2022 and June 2023 at pulmonary outpatient clinics in Türkiye (N=397)

*Characteristics*	*Total (N=397) n (%)*	*Usual support (N=199) n (%)*	*Immediate support (N=198) n (%)*	*p*
**Airway disease**				0.266
COPD	220 (55.4)	106 (57.6)	114 (57.6)	
Asthma	165 (41.5)	89 (44.7)	76 (38.4)	
Bronchiectasis	12 (3.02)	4 (2.0)	8 (4.0)	
**Age** (years), mean (SD)	53.5 (13.1)	54.4 (13.1)	52.7 (13.1)	0.251
**Sex**				0.144
Female	130 (32.7)	72 (36.2)	58 (29.3)	
Male	267 (67.2)	127 (63.8)	140 (70.7)	
**Marital status**				0.096
Married	321 (80.8)	154 (77.4)	167 (84.3)	
Single	50 (12.5)	27 (13.6)	23 (11.6)	
Separated	26 (6.54)	18 (9.0)	8 (4.0)	
**BMI** (kg/m^2^), mean (SD)	26.8 (4.74)	27.0 (5.03)	26.7 (4.45)	0.738
**Education level**				0.003
Primary schooling	177 (44.5)	99 (49.7)	78 (39.4)	
Secondary schooling	63 (15.8)	34 (17.1)	29 (14.6)	
High school graduate	95 (23.9)	48 (24.1)	47 (23.7)	
University graduate	62 (15.6)	18 (9.0)	44 (22.2)	
**Income level**				0.185
≤Minimum wage	246 (62.0)	122 (61.3)	124 (62.6)	
≤3 times the minimum wage	112 (28.2)	62 (31.2)	50 (25.3)	
>3 times the minimum wage	39 (9.8)	15 (7.5)	24 (12.1)	
**Household member number**, mean (SD)	3.18 (1.58)	3.16 (1.44)	3.19 (1.70)	0.578
**Occupation**				0.214
Retired	122 (30.7)	64 (32.2)	58 (29.3)	
Housewife	80 (20.2)	46 (23.1)	34 (17.2)	
Employed	161 (40.6)	76 (38.2)	85 (42.9)	
Unemployed	34 (8.6)	13 (6.5)	21 (10.6)	
**Diagnosis duration** (years), mean (SD)	6.21 (7.09)	6.20 (7.10)	6.21 (7.11)	0.709
**Initiation age to smoking** (years), mean (SD)	19.2 (6.78)	19.2 (6.93)	19.3 (6.65)	0.777
**Number of cigarettes smoked daily**, mean (SD)	20.8 (10.9)	19.1 (9.78)	22.5 (11.7)	0.002
**Number of years smoking**, mean (SD)	32.7 (14.9)	33.5 (15.1)	32.0 (14.8)	0.383
**Smoking pack-years**, mean (SD)	37.8 (25.5)	36.8 (24.3)	38.7 (26.7)	0.739
**Fagerström test for nicotine dependence score**, mean (SD)	6.14 (2.82)	5.78 (2.74)	6.50 (2.85)	0.011
**Presence of depression diagnosis**	68 (17.1)	36 (18.1)	32 (16.2)	0.610
**Presence of anxiety diagnosis**	75 (18.9)	31 (15.6)	44 (22.2)	0.091
**Presence of other comorbidities**	191 (48.1)	104 (52.3)	87 (43.9)	0.097
**FEV1/FVC** %, mean (SD)	73.0 (13.2)	72.4 (12.8)	73.7 (13.6)	0.166
**FEV1** %, mean (SD)	69.1 (22.2)	66.2 (21.5)	72.0 (22.6)	0.039
**GOLD categories of COPD patients**				0.980
A	65 (29.5)	31 (29.2)	34 (29.8)	
B	90 (40.9)	43 (40.6)	47 (41.2)	
E	65 (29.5)	32 (30.2)	33 (28.9)	
**Asthma severity**				0.060
Mild	83 (50.6)	39 (44.3)	44 (57.9)	
Moderate	73 (44.5)	42 (47.7)	31 (40.8)	
Severe	8 (4.9)	7 (8.0)	1 (1.3)	
**Unscheduled doctor visits in last year**, mean (SD)	0.88 (1.33)	0.74 (1.29)	1.02 (1.36)	0.003

COPD: chronic obstructive pulmonary disease. BMI: body mass index. FEV1: forced expiratory volume in first second. FVC: forced vital capacity. Following tests were used for analysis: for continuous variables Student’s t-test, for categorical variables chi-squared test.

Comparisons according to the allocation arm are also presented in Table 1. When comparing the immediate support arm to the usual care arm, several notable differences were observed. Compared to the usual care arm, the proportion of patients with a university education was higher, and the proportion of patients with a primary school education was lower in the immediate support arm. The mean number of cigarettes smoked daily and mean FTND scores were higher in the immediate support arm. In addition, in the immediate support arm, the mean FEV1% predicted and the mean unscheduled doctor visit numbers were also higher (p<0.05).

**Figure 1:**
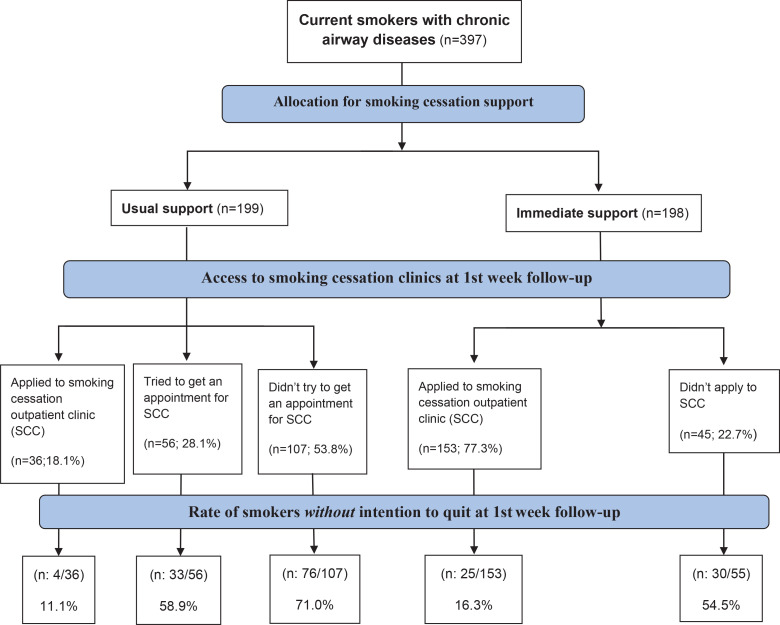
Flow diagram of the randomized patients. Multicenter, prospective, randomized, open-label study conducted between November 2022 and June 2023 at pulmonary outpatient clinics in Türkiye (N=397)

A total of 397 patients were enrolled in the study, with 199 allocated to the usual support arm and 198 allocated to the immediate support arm. Within the first week, 18.1% of patients in the usual support arm and 77.3% of patients in the immediate support arm sought assistance from the smoking cessation clinic (p<0.001). Among those in the usual support arm, 113 out of 199 (56.7%) reported no quit effort at the end of the first week, while in the immediate support arm, this rate was 55 out of 198 (27.7%). Notably, the highest rate of individuals who did not make an effort to quit smoking was observed among those in the usual support arm who did not schedule an appointment at the smoking cessation outpatient clinic or were unable to secure an appointment (71% and 58.9%, respectively) (Figure 1).

Table 2 represents the logistic regression model of covariates associated with admission to smoking cessation clinic within the first week. The model was adjusted for income level, FEV1%, FTND score, airway disease type and education level. Immediate support arm was associated with admission to the cessation clinic, being approximately 13 times more likely than usual care (AOR=13.38; 95% CI: 8.00–22.38).

**Table 2:** Multivariable logistic regression analysis of associated factors with access to smoking cessation clinics. Multicenter, prospective, randomized, open-label study, conducted between November 2022 and June 2023 at pulmonary outpatient clinics in Türkiye (N=397)

*Variables*	*β*	AOR*	*95% CI*	*p*
**Randomization arm**				
Usual support	Ref.			
Immediate support	2.594	13.38	8.00–22.38	<0.001
**Age** (years) (per 1 unit age increase)	0.015	1.02	0.96–1.08	0.624
**Presence of comorbidity**				
Present	Ref.			
Absent	0.302	1.35	0.76–2.41	0.305
**Duration of smoking** (years) (per 1 year increase)	-0.037	0.96	0.91–1.02	0.209
**Initiation age of smoking** (years) (per 1 year increase)	-0.026	0.975	0.91–1.04	0.460
**Anxiety disorder**				
Present	Ref.			
Absent	-0.439	0.64	0.33–1.28	0.207

AOR: adjusted odds ratio. FEV1: forced expiratory volume in 1st second.

* Model is adjusted for income level, FEV1%, Fagerström test score, types of airway diseases, and education level.

## DISCUSSION

Our study findings suggest that immediate appointment scheduling in smoking cessation clinics for patients with chronic airway disease who are current smokers during routine outpatient service can significantly enhance the likelihood of applying to the smoking cessation outpatient clinic. Comparing the immediate appointment group to the usual care group, we observed a substantial difference in clinic utilization, with a significantly higher rate in the immediate appointment group (77% vs 18%). Moreover, even after adjusting for confounding factors, our results indicate a strong and positive association between immediate appointment scheduling and referrals to the smoking cessation clinic, with a 13-fold higher likelihood compared to routine service. These findings underscore the potential benefits of promptly implementing immediate appointment scheduling as an effective approach to support smoking cessation efforts among patients with chronic airway disease.

Smoking cessation outpatient clinic services have been widely established nationwide, demonstrating exemplary practice and providing access to free smoking cessation pharmacological treatments^8^. Currently, there are over 300 smoking cessation outpatient clinics, primarily facilitated by pulmonary physicians, accounting for 57.5%^9^. However, it is noteworthy that tobacco control policies do not uniformly incorporate smoking cessation services or programs across all healthcare facilities. Similar challenges have been reported in other low- to middle-income countries, such as inadequate training, time constraints, and a limited number of health professionals available to deliver these services, further contributing to these barriers^15^.

The ongoing quest for solutions aims to enhance evidence-based smoking cessation assistance and address the aforementioned obstacles. An emphasized novel approach is the integration of tobacco cessation care into routine practice^16^. Physicians face significant challenges in accessing smoking cessation support, necessitating a comprehensive understanding of the current landscape and potential solutions. As pulmonary physicians, our objective was to investigate the effects of two distinct smoking cessation interventions on cessation attempts among patients with chronic airway diseases identified during the routine outpatient clinics.

Smoking cessation pharmacological treatments are typically prescribed during quit attempts, but they can also be initiated during the smoking reduction process and, thus, the preparation for quitting. Randomized trials have reported higher quit rates with immediate and effective cessation interventions, regardless of willingness to quit smoking, compared to planned quit attempts^17^. Smokers with chronic airway diseases can access smoking cessation services through appointment-based quitlines, similar to the general population, and their quit rates have been found to be comparable^1,2^. Nevertheless, it is well documented that tobacco cessation support for chronic airway diseases remains a neglected issue, not only among pulmonologists but also among other physicians^18^. A multinational qualitative study identified underlying reasons for this, highlighting physicians’ general issues and attitudes towards cessation treatment and the management of patients with COPD. These issues encompassed frustration, inexperience, and stigmatization among physicians regarding smoking cessation in their COPD patients and in the general smoking population. Furthermore, financial and time constraints were also identified as barriers for physicians^18^.

In order to increase the motivation of patients with chronic airway disease diagnosis who continue to smoke, such as our sample, personalized interventions may be more effective in engaging them with smoking cessation centers. Therefore, such unmotivated groups may need more intensive and individualized interventions to support their involvement in smoking cessation programs^19^. A recent study found no significant difference in motivation levels to quit between individuals who voluntarily desired to quit smoking and those who attempted to quit with the advice of a healthcare professional^20^. Healthcare providers play a crucial role in motivating their smoker patients and implementing evidence-based tobacco cessation methods. However, despite that over 70% of adults expressed a desire to quit, less than half received cessation advice in the past year, and only 30% had access to evidence-based quitting methods. Clinicians can help patients to quit smoking when they are diagnosed with tobacco-related disease, also known as the ‘teachable moments,’ or by using the moments during hospitalization effectively^21^. The highest rate of quitting success in quitting occurs when physicians strongly implement the 5As model of brief quitting advice and provide access to evidence-based cessation methods^22^. A systematic review revealed that 65% of medical staff ‘Ask’ 63%, ‘Advise’ 36%, ‘Assess’ 44%, ‘Assist’ and 22% ‘Arrange’ in terms of smoking cessation support^23^. Additionally, healthcare providers tend to overlook providing cessation support to patients with chronic airway diseases^24^. In our study, both groups underwent ‘asking, advising, assessing, and assisting’, with one group receiving more intensive arrangements and the other group being informed about appointment scheduling. The application rate was 73% in the more intensive arrangement group, compared to 18% in the other group. Thus, the immediate arrangement arm displayed higher motivation for quitting.

Among the factors contributing to deficiencies in healthcare providers’ ability to address smoking cessation, several have been identified. These include inadequate time, insufficient training and self-efficacy in tobacco addiction treatment, and limited staff availability. Additionally, healthcare providers often express a lack of necessary resources and information to determine the appropriate referral services. From the perspective of patients, there are various potential barriers to smoking cessation support. These include extended waiting periods, such as the average waiting time of 29.3 days to secure an appointment with a primary care provider for a smoking cessation prescription. Other obstacles reported include the distance patients must travel to access a provider and the limited availability of doctor’s appointments outside of typical office hours^22^. A national study focusing on access to smoking cessation outpatient clinics highlighted transportation difficulties faced by individuals, emphasizing the need for improved accessibility^25^. An evaluation of smoking cessation practice, barriers, and facilitators of healthcare providers in Mexico evaluated several barriers, including patient motivation, time constraints for assessment, long waiting-times for appointments, and insufficient training. Reported facilitators include the existence of cost-free smoking cessation programs, access to pharmacotherapy, and the presence of multidisciplinary teams^15^.

### Strengths and limitations

This study was predominantly conducted by pulmonologists who specialize in managing patients with chronic airway diseases. As a result, there is a high level of agreement regarding diagnosis accuracy, follow-up, and patient allocation. Furthermore, the study addresses the often-overlooked issue of ‘smoking cessation support’ by physicians, offering valuable insights that can potentially serve as a solution. However, the limitations of the study include the inability to achieve full homogeneity in the distribution across all variables due to the sequential allocation in addition to residual confounding, and limited generalizability to other countries. Nonetheless, the study still includes a sufficient number of cases to demonstrate the significant benefits of the immediate support program in various healthcare settings and among patients with diverse characteristics.

## CONCLUSIONS

Our study strongly recommends immediate appointment scheduling for smoking cessation outpatient clinics as an effective strategy to increase clinic utilization among current smokers with chronic airway disease. Integrating comprehensive smoking cessation interventions into routine clinical practice and establishing supportive infrastructures within healthcare institutions are crucial. Pulmonary disease specialists should play an active role in providing smoking cessation services to improve quit rates and reduce the burden of tobacco-related diseases. Further research and implementation efforts are needed to address barriers and enhance access to evidence-based quit methods in healthcare settings. These measures can significantly improve patient outcomes and promote healthier lifestyles for current smokers with chronic airway disease.

## Data Availability

The data supporting this research are available from the authors on reasonable request.
